# Supplementary material for: Psychometric properties of three online-related addictive behavior instruments among Bangladeshi school-going adolescents
Source: PLoS One. 2022 Dec 14;17(12):e0279062. doi: 10.1371/journal.pone.0279062 (PMC9750012; doi:10.1371/journal.pone.0279062)
Supplement: S2 File — (DOCX) [file pone.0279062.s002.docx]

**Table 1:** Mean differences and associations of the three scales with socio-demographic variables

| **Variables** | **IGDS9-SF score** | | | **GDT score** | | | **BSMAS score** | | |
| --- | --- | --- | --- | --- | --- | --- | --- | --- | --- |
|  | Mean (SD) | t/F | *p*-value | Mean (SD) | t/F | *p*-value | Mean (SD) | t/F | *p*-value |
| **Sex** |  |  |  |  |  |  |  |  |  |
| Male | 19.33 (7.58) | 1.13 | 0.289 | 8.28 (3.87) | 1.28 | 0.259 | 14.53 (6.38) | 0.90 | 0.343 |
| Female | 20.72 (9.58) |  |  | 7.54 (4.1) |  |  | 15.54 (5.25) |  |  |
| **Marital status** |  |  |  |  |  |  |  |  |  |
| Unmarried | 19.15 (7.74) | 3.39 | 0.035 | 8.17 (3.88) | 4.18 | 0.016 | 14.27 (6.22) | 10.86 | <0.001 |
| Married | 24.55 (11.3) |  |  | 11.45 (4.82) |  |  | 11.36 (2.94) |  |  |
| In a relationship | 20.86 (6.63) |  |  | 7.77 (3.52) |  |  | 18.53 (6.08) |  |  |
| **Academic grades** |  |  |  |  |  |  |  |  |  |
| Secondary | 18.5 (6.58) | 25.31 | <0.001 | 7.72 (3.4) | 26.48 | <0.001 | 14.2 (6.26) | 7.54 | 0.006 |
| Higher secondary | 23 (10.46) |  |  | 10.02 (4.96) |  |  | 16.22 (6.17) |  |  |
| **Family type** |  |  |  |  |  |  |  |  |  |
| Nuclear | 19.86 (7.99) | 3.55 | 0.060 | 8.4 (3.94) | 3.12 | 0.078 | 14.99 (6.53) | 4.52 | 0.034 |
| Joint | 18.21 (6.99) |  |  | 7.62 (3.7) |  |  | 13.49 (5.35) |  |  |
| **Monthly income** |  |  |  |  |  |  |  |  |  |
| <15,000 BDT | 21.2 (8.4) | 4.11 | 0.007 | 9.04 (4.12) | 3.02 | 0.030 | 15.54 (6.06) | 1.19 | 0.313 |
| 15,000-30,000 BDT | 17.94 (6.72) |  |  | 7.69 (3.61) |  |  | 14.27 (5.84) |  |  |
| 30,000-45,000 BDT | 18.47 (6.31) |  |  | 7.61 (2.97) |  |  | 13.85 (6.2) |  |  |
| >45,000 BDT | 20.46 (8.77) |  |  | 8.6 (4.38) |  |  | 14.94 (6.77) |  |  |
| **Living status** |  |  |  |  |  |  |  |  |  |
| With parents | 19.07 (7.59) | 4.73 | 0.009 | 7.94 (3.74) | 8.98 | <0.001 | 14.56 (6.31) | 0.22 | 0.805 |
| Only father or mother | 21.6 (5.92) |  |  | 10 (3.95) |  |  | 15.4 (4.67) |  |  |
| Without parents | 23.68 (11.11) |  |  | 10.86 (5.01) |  |  | 14.82 (7.55) |  |  |

Table 2: Correction of age with the three scales

| **Variables** | **1** | **2** | **3** |
| --- | --- | --- | --- |
| 1. Age | 1.00 | 0.14^**^ | 0.18^**^ |
| 2. IGDS9-SF | 0.14^*^ | 1.00 | 0.71^**^ |
| 3. GDT | 0.18^**^ | 0.71^**^ | 1.00 |
| 4. BSMAS | 0.18^**^ | 0.31^**^ | 0.24^**^ |

*Note:* **p*<0.01, ***p*<0.001
