# Supplementary material for: Psychometric properties of three online-related addictive behavior instruments among Bangladeshi school-going adolescents
Source: PLoS One. 2022 Dec 14;17(12):e0279062. doi: 10.1371/journal.pone.0279062 (PMC9750012; doi:10.1371/journal.pone.0279062)
Supplement: Attachment — Submitted filename: Review Plos One.docx [file pone.0279062.s003.docx]

PLOS ONE _ Peer review

**Title: Psychometric properties of three online-related addictive behavior instruments among Bangladeshi school-going adolescents**

The presentation of the validation of the adapted versions of three scales belonging to the topic of internet addiction is to be appreciated. Beyond these, there are a number of methodological issues, more precisely certain aspects that do not consistently support what the authors claim. Find the related details below:

(1) The theoretical framing of the research is missing. The relevant theories for the behavioral addictions analyzed in this research should be mentioned.

(2) Previous studies in the psychometric literature regarding versions adapted in other languages/cultures are also missing. A few are only mentioned in the Discussions section. To complete these aspects and to emphasize the importance/necessity of the extension of this empirical body, an extension to which you are actually trying to contribute.

(3) It should also be mentioned that the validation studies include CTT, as well as advanced approaches (i.e., IRT).

For IGD9SF mention:

<https://doi.org/10.1007/s11469-018-9890-z>

For BSMAS mention:

<https://doi.org/10.1556/2006.6.2017.071>

<https://doi.org/10.1007/s11469-021-00732-7>

Also mention the studies that proposed various cutoffs:

For IGD9SF

<https://doi.org/10.3389/fpsyt.2020.00470>

For BSMAS

<https://doi.org/10.1556/2006.2021.00025>

Even if you mentioned some of them (not all) in the Discussions, they must be specified (synthetically, obviously) in the theoretical background as well

(4) Page 8, line 3: It should be mentioned that the adapted versions of the Patient Health Questionnaire [PHQ-9] and Generalized Anxiety Disorder [GAD-7] questionnaires were used).

(5) You did not mention anything about outliers, multivariate data distribution, estimation method used, in CFA

(6) You analyzed the reliability using the Cronbach alpha coefficient, although the tau-equivalence assumption is clear that it is not fulfilled because all item discriminations are not equal. To be completed here with model-based reliability, which does not require tau-equivalence

(7) You did not explain the connection between floor effect and ceiling effect with psychometric robustness. More precisely, you emphasized that these effects are not present (although in the case of one scale the cutoff recommended in the literature was slightly exceeded), but you did not explain which is relevant to the validity of the studied scales

(8) According to the data presented in Table 6, it is a problem related to the IGD9SF scale. From what you report, it appears that only 34% of the variance is explained by the modeled latent variable, that is, internet gaming disorder.

You did not correctly reproduce the amendment proposed by Fornell and Larker (1981). They specified that AVE has a cutoff >.50, but a threshold of .40 is accepted only if CR>.60. You mentioned something else on page 15, namely that it is about values lower than .50, although the authors explicitly mention that the threshold of .40 is also accepted. It can be seen that this criterion is not met in your case, since AVE is .34.

(9) You did not discuss anything about the fact that a third of the items of this scale have small slopes, slightly above the allowed threshold. Obviously, that is why the AVE is so small.

(10) You correlated the residuals in three situations and did not explain anything. Anyway, this option is considered in the psychometric literature to be avoided because it represents an artificial forcing to obtain a good model fit.

To inspect the covariance matrix

(11) It should be mentioned that the RMSEA in the case of BSMAS indicates a mediocre fit between your model and the data

(12) Page 17 _ You stated that: "In the present study, all fit indices obtained in the CFA were well-accepted in their conventional thresholds". As I mentioned above, it is to be discussed here. The term “all” is too inclusive compared to the results obtained. So reformulate, nuance and explain.

Good luck!
